# Vascular endothelial growth factor-A and drug level in serum and human breast milk of a lactating woman after intravitreal injection of ranibizumab: a case report

**DOI:** 10.3389/fmed.2026.1730208

**Published:** 2026-01-30

**Authors:** Jiaxuan Zhang, Bojia Niu, Yu Wang, Yiwei Li, Qi Wang, Ying Xiao

**Affiliations:** Department of Ophthalmology, Shandong Provincial Hospital Affiliated to Shandong First Medical University, Jinan, Shandong, China

**Keywords:** case report, enzyme-linked immunosorbent assay, human breast milk, ranibizumab, vascular endothelial growth factor-A

## Abstract

**Background:**

Evidence on the excretion of anti-VEGF drugs in human breast milk was very limited. Here we investigated the level of VEGF-A protein and free drug concentration of ranibizumab in serum and breast milk following intravitreal injection of ranibizumab in a young woman suffering from branch retinal vein occlusion (BRVO).

**Case presentation:**

A 30-year-old female, who was nursing her six-month-old son, was treated by intravitreal injection of ranibizumab (IVR) for cystoid macular edema owing to BRVO of her right eye. Serum and breast milk samples were collected before and within 4 weeks after injection. Levels of VEGF-A and free ranibizumab were evaluated in all the samples by the enzyme-linked immunosorbent assay (ELISA). Ranibizumab concentration in both serum and breast milk increased rapidly on day one, with ranibizumab level in serum peaked at 15.35 ng/mL and ranibizumab level in breast milk peaked at 3.14 ng/mL. The VEGF-A protein remained a very low level in serum, while decreased over the first three days from 29.49 ng/mL to 12.75 ng/mL, then slowly increased to almost pre-injection level in breast milk.

**Conclusion:**

Systemic exposure to ranibizumab was observable following intravitreal injection in a breastfeeding patient. While serum levels of VEGF-A remained stable following ranibizumab administration, the functional alterations resulting from variations in VEGF-A in breast milk and the implications for breastfeeding infants were ambiguous. When lactating women experience specific sight-threatening conditions, additional evaluations are required to ascertain the safety profile of anti-VEGF therapy for nursing infants.

## Introduction

In recent years, anti-vascular endothelial growth factor (VEGF) agents, including ranibizumab, bevacizumab, aflibercept and Faricimab, have emerged as the primary treatment for various retinal disorders. Lactating women may necessitate prompt intravitreal injections of anti-VEGF agents for visually threatening retinal conditions, including choroidal neovascularization (CNV), diabetic macular edema, or macular edema secondary to retinal vein occlusion. Human breast milk contains various growth factors including VEGF-A (1), which is critical for the development of infant lungs and intestines. However, reports concerning anti-VEGF treatment in lactating women are rare (2–5). The limited knowledge on the excretion of anti-VEGF agents in human breast milk raised concerns about the safety of intravitreal injection of these drugs in nursing mothers and their babies.

In this study, we enrolled a lactating woman with branch retinal vein occlusion (BRVO) in the right eye who received intravitreal administration of 0.5 mg ranibizumab (Lucentis; Genentech Inc., South San Francisco, CA, US). Blood and breast milk were collected within 4 weeks after intravitreal injection. Concentrations of VEGF-A as well as ranibizumab at multiple timepoints were evaluated to provide more information on the impact of anti-VEGF medication on nursing women and their newborns.

## Case report

A 30-year-old lactating woman was admitted to Shandong Provincial Hospital with complaint of sudden vision decrease and metamorphopsia in the right eye for 1 week. The patient had a one-year history of hypertension and family history of open-angle glaucoma. Best corrected visual acuity was 0.3 OD. Intraocular pressure was normal for both eyes. Retinal venous engorgement in the supratemporal quadrant, as well as retinal hemorrhages were detected by fundus examination. Macular edema secondary to BRVO was proved by OCT. Her left eye was normal. Intravitreal injection of ranibizumab (IVR) 0.5 mg/0.05 mL in the right eye was performed. No side effect happened after IVR.

Blood and breast milk samples of the patient were collected 1 h prior to IVR (baseline) and at 2 h, 6 h, 1 day, 3 days, 7 days, 14 days, and 28 days after IVR therapy. After collection, the blood samples were centrifuged at 3000 rpm and 4 °C for 10 min. The breast milk samples were centrifuged at 13000 rpm and 4 °C for 15 min. All the supernatants were transferred to sterile tubes and stored at −80 °C.

Concentration of VEGF-A and free ranibizumab in serum and breast milk were determined by employing enzyme-linked immunosorbent assay (ELISA) (Human Vegf Elisa Kit, Boster, Wuhan, China; Ranibizumab ELISA Kit, Matriks Biotechnology, Turkey). Informed consent form was received from the patient prior to the study. The lower limit of quantification (LLOQ) of VEGF-A and ranibizumab were 31.2 pg./mL and 0.3 ng/mL, respectively. Samples were run in triplicate for analysis. The average value and standard deviation (SD) were calculated. The intra-assay coefficients of variation (CV) were derived from the authors’ own lab experiments. CV for all measurements in this study was below 15%, which was within the acceptable range for ELISA-based assays and indicated excellent reproducibility of our measurements.

**Figure 1:**
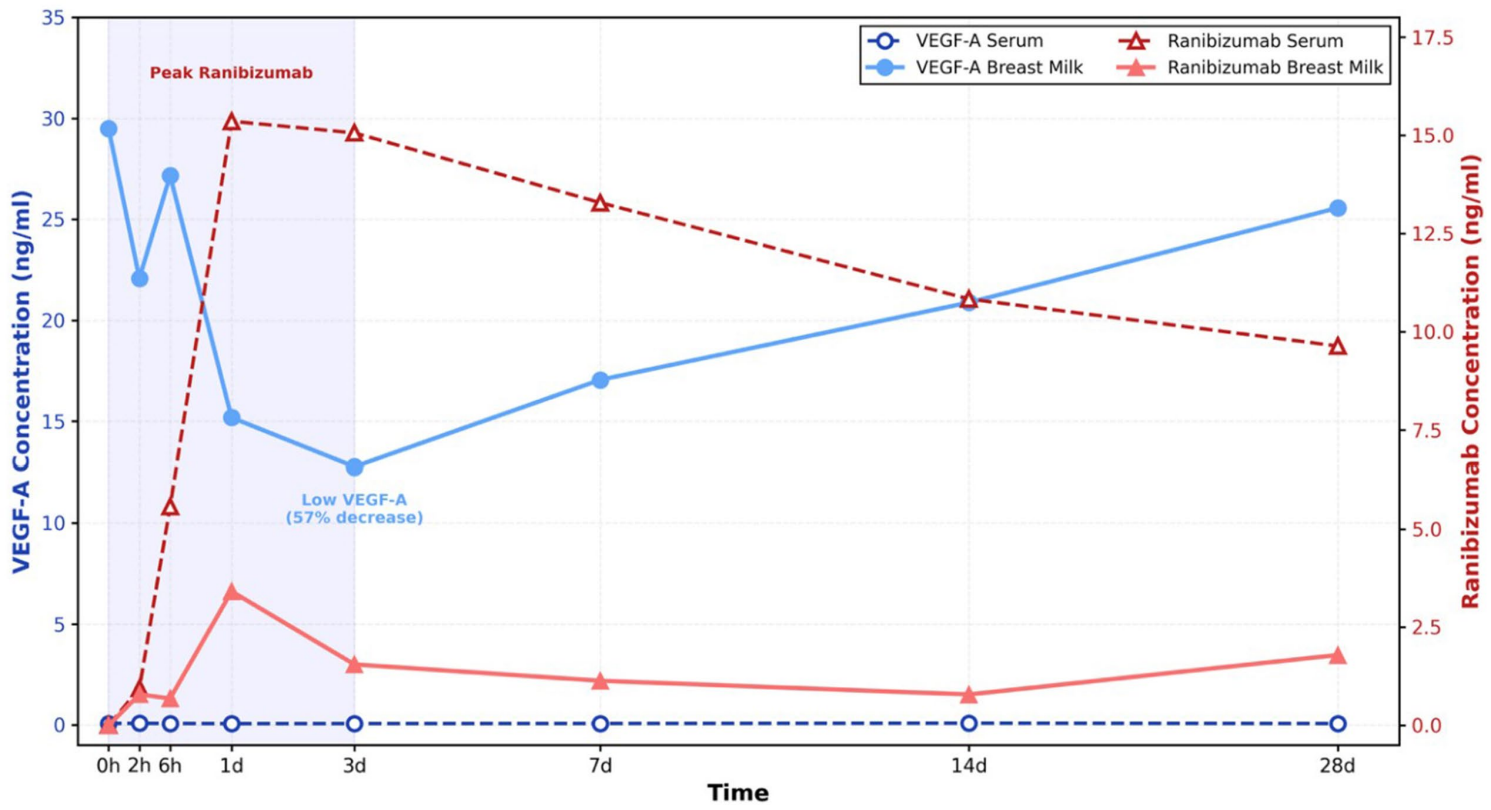
Ranibizumab and VEGF-A concentration post intravitreal injection. Changes of VEGF-A and ranibizumab concentration over a logarithmic time scale. Ranibizumab concentration in both serum and breast milk increased rapidly on day one, with ranibizumab level in serum peaked at 15.35 ng/mL and ranibizumab level in breast milk peaked at 3.14 ng/mL. The VEGF-A concentration in breast milk decreased over the first three days and then slowly increases to almost pre-injection level.

Concentration of VEGF-A and ranibizumab in serum and breast milk were presented in Figure 1. Free drug concentration of ranibizumab was undetectable in both serum and breast milk prior to intravitreal injection of ranibizumab (IVR). Rapid rise of ranibizumab in serum was verified by ELISA following IVR. Maximum concentration was observed on day 1 at 15.357 ng/mL and sustained elevated levels throughout the first week. Ranibizumab remained present in serum throughout the study duration, with concentration decreasing to 9.635 ng/mL by week 4. The concentration of the free drug in breast milk was significantly lower than that in serum. The maximum concentration of ranibizumab in breast milk was observed on the first day, but it was approximately one-fifth of the serum concentration. It exhibited a decrease from 3.414 ng/mL on day 1 to 1.793 ng/mL on day 28.

During the full 4 weeks, VEGF-A protein remained a very low level in serum for no more than 0.1 ng/mL. In breast milk, VEGF-A displayed the maximum concentration of 29.491 ng/mL before IVR. After anti-VEGF injection, VEGF-A declined significantly from day 1 and reached the lowest concentration of 12.748 ng/mL at the 3rd day, which was 43% of the starting status. After that, it grew progressively and reverted to 25.56 ng/mL, the level close to the baseline at week 4.

After carefully considering the potential risks associated with ranibizumab secreted into the breast milk and the benefits of breastfeeding for the infant, the patient made a decision to stop breastfeeding for 1 week after IVR. Breast milk was pumped out and discarded during this period. After 1 week, she resumed nursing the baby but reduced the frequency of breastfeeding and introduced infant supplementary food to the infant. 4 weeks after IVR, BCVA of the right eye was 0.9. Macular edema was totally regressed. No more injection was performed from then on. No abnormalities in infant growth and development were observed during and after the study period.

## Discussion and conclusion

Currently, most patients necessitating anti-VEGF medication are over the age of 50. Nonetheless, anti-VEGF medicines may be advantageous for younger individuals, including certain reproductive women afflicted with CNV or other retinal vascular disorders. Considering the potential risks of any medication to pregnant women, numerous participants have discontinued ocular treatment despite significant vision impairment. In addition, the extent of anti-VEGF medication transfer from the vitreous to breast milk following intraocular injection remains largely unknown. It is recommended that nursing be discontinued during anti-VEGF therapy due to probable adverse effects on the newborn. Further research is required to precisely measure the alterations in VEGF and the medication concentration in blood and breast milk to meet the specific demands of nursing women and their infants.

As we know, systemic exposure of anti-VEGF drugs after intravitreal injection are closely related with their molecular structures and properties. All the Anti-VEGF agents can rapidly move into blood stream and produce a reduction in plasma free VEGF. Fc-containing molecules, such as bevacizumab and aflibercept, are recycled by binding endothelial cell FcRn receptors to protect them from the degradation and decrease the rate of systemic clearance. However, Ranibizumab is a 48-kilodalton protein exclusively consisting of monoclonal antibody antigen-binding fragment (Fab). Although it can rapidly transfer across the blood-retina barrier and subsequently secret into circulation, the lack of the crystallizable fragment (Fc) region facilitates its rapid clearance from the systemic circulation and dramatically shorten its systemic half-life (6, 7). Among all the anti-VEGF agents, ranibizumab is the only one which has been approved for intravitreal treatment of retinopathy of prematurity (ROP), representing its comparative safety for infants (8). No developmental abnormalities were reported in the treated premature infants within a two-year period (9). Based on the pharmacokinetics profile, ranibizumab was chosen for the patient’s treatment in our study because of its relatively short half-life to other VEGF inhibitors, hence minimizing the systemic accumulation and reducing potential exposure to the infant.

Several studies have indicated that anti-VEGF drugs may permeate breast milk through circulation and diminish VEGF levels in both blood and milk, but ranibizumab exerts a lesser effect on VEGF concentration compared to bevacizumab or aflibercept (3, 4, 10). Agostini et al. reported a significant reduction in VEGF levels in both plasma and human breast milk for approximately 8 weeks following the intravitreal injection of bevacizumab. However, VEGF levels in plasma and breast milk reverted to baseline concentrations within one week following the patient’s transition on ranibizumab (3). Avery et al. demonstrated that both ranibizumab and aflibercept are present in breast milk, accompanied by a decrease of VEGF-A following intravitreal injection (4). Lin et al. recently reported that the concentration of VEGF-A in human breast milk initially decreased and subsequently returned to pre-injection levels one day post-IVR (5). For another kind of anti-VEGF multitarget drug, conbercept, VEGF concentrations in the breast milk samples slightly decreased in one lactating woman during the first week, while kept stable in another two women’s breast milk samples (2).

In this case, after intravitreal injection of ranibizumab, the antibody may traverse the blood-retina barrier, entering the bloodstream before being discharged into breast milk. Consequently, the concentration of the free medication in the serum would be significantly greater than that in the milk. In this study, the maximum concentration of ranibizumab was observed on the first day in both serum and breast milk, aligning with prior pharmacokinetic results (11). Although the patient ceased breastfeeding within the first week post-injection, she pumped milk throughout this period, which prevented sustained accumulation of the anti-VEGF agent in the breast milk.

Given that children assimilate antibodies from breast milk, we hypothesize that the infant could ingest and absorb a portion of free ranibizumab antibody from the maternal milk. At every time point, the concentration of free ranibizumab in breast milk was considerably lower than the *in vitro* IC50 values for VEGF based on the bovine retinal microvascular endothelial cell proliferation assay (11–27 ng/mL) (7). According to RAINBOW research, the maximal serum concentration of ranibizumab neared the IC50 range following intravitreal injection into both of the newborn’s eyes. However, there was no discernible decrease in plasma-free VEGF levels, nor any negative impacts on the growth or neurodevelopment of infants (8). In addition, the infant of our patient was 6 months old, indicating a lower intestinal permeability and a better-established intestinal system than that of a newborn. As a result, we believe it is unlikely that a newborn exposed to ranibizumab through breastfeeding will have serious adverse effects. To evaluate the impact of free anti-VEGF agents on breastfed newborns, more observation is necessary.

In this investigation, serum VEGF protein levels in participants were stable, consistently measuring no more than 0.1 ng/mL following IVR. Clinical study has indicated that the baseline concentration of serum VEGF varies significantly, ranging from 10 to 600 pg./mL, depending on the population and methodology employed (7, 12, 13). An identical outcome was achieved if ranibizumab exerted no substantial influence on serum VEGF concentration, aligning with our findings. Regarding VEGF-A level in breast milk, it was much higher than that in serum in our case. A discernible decline of approximately 57% from baseline levels was seen in breast milk due to IVR. The minimal concentration was observed on day 3. We hypothesize that this may result from the highest concentration of ranibizumab in breast milk on day one. Ranibizumab elicited a concomitant decrease in VEGF levels via binding to free VEGF-A found in the breast milk of lactating women. VEGF-A is synthesized by lactiferous mammary gland epithelial cells and is seen in elevated amounts in human breast milk (14, 15). It is a bioactive element that primarily regulates fetal vascular angiogenesis and facilitates the growth, maturation, and maintenance of the neonatal gastrointestinal tract. Dysregulation of VEGF-A may impair microvascular development, leading to organ dysfunction and increased morbidity. Growing evidence suggests that reduced VEGF levels are associated with bronchopulmonary dysplasia, pulmonary hypertension, and necrotizing enterocolitis (16, 17). Despite the temporary fluctuations in milk VEGF levels observed in our case, the potential for adverse effects in the newborn due to decreased VEGF-A cannot be dismissed. The administration of intravitreal VEGF inhibitors is contraindicated during pregnancy and lactation. Ranibizumab should be prioritized or recommended if anti-VEGF therapy is necessary. Our research offers valuable insights to ophthalmologists and lactating patients seeking to mitigate the dangers of anti-VEGF drugs to their breastfeeding infants while undergoing intravitreal injections for vision-threatening conditions.

This study has some limitations. First, control samples of breast milk and serum were not obtained from the healthy lactating individuals or other nursing mothers who also received IVR treatment. So, a comparison could not be conducted between different individuals regarding drug metabolism. Another limitation is that serum or plasma tests were not performed on the infant due to the invasive nature of the procedures. Consequently, data about alterations in ranibizumab and VEGF-A levels in the child could not be acquired. Additionally, as a single case study, this research only presented limited data on drug clearance and VEGF fluctuation in a lactating woman. It may only function as a reference for analogous patients. Definitive conclusions still require validation through studies with larger sample sizes.

In conclusion, ranibizumab was detectable in both serum and breast milk following intravitreal injection in a nursing woman. While blood VEGF-A levels remained steady following ranibizumab treatment, the functional effects on the breastfeeding infant due to the brief fluctuations of VEGF-A in human breast milk cannot be determined. Additional research is required to assess the safety profile of anti-VEGF medication for nursing infants when lactating patients are afflicted with certain sight-threatening conditions.

## Data Availability

The raw data supporting the conclusions of this article will be made available by the authors, without undue reservation.
