# Supplementary figures and images for: Vascular endothelial growth factor-A and drug level in serum and human breast milk of a lactating woman after intravitreal injection of ranibizumab: a case report
Source: Front Med (Lausanne). 2026 Jan 30;13:1730208. doi: 10.3389/fmed.2026.1730208 (PMC12900737; doi:10.3389/fmed.2026.1730208)

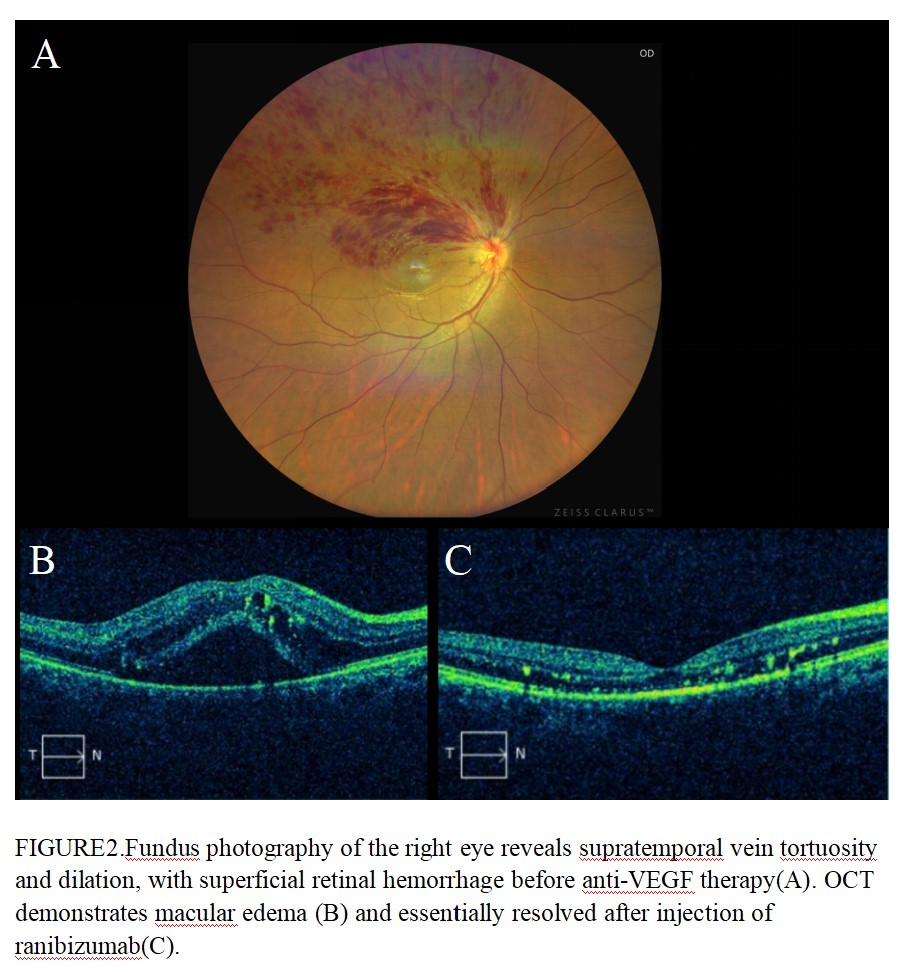

Supplement: Supplementary file 1 [file Image_1.JPEG]

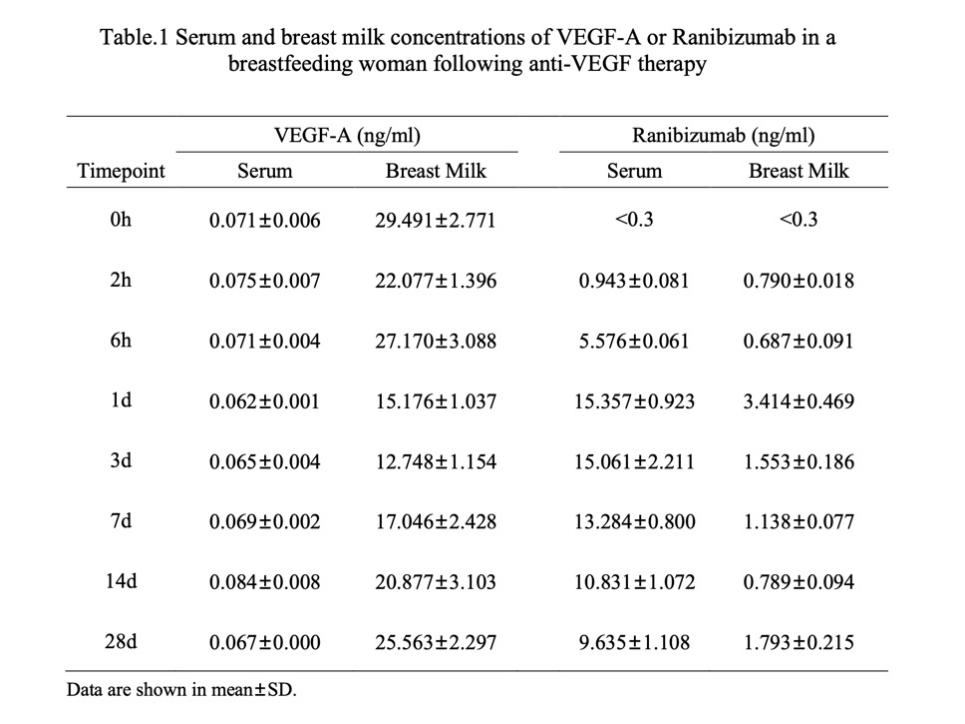

Supplement: Supplementary file 2 [file Image_2.JPEG]
